# A Phase I/II Study of a 72-h Continuous Infusion of Etoposide in Advanced Soft Tissue Sarcoma

**DOI:** 10.1080/13577149778236

**Published:** 1997-12

**Authors:** Charles R. Crawley, Ian R. Judson, Mark Verrill, Catherine Hill, Florence I. Raynaud

**Affiliations:** 1 ICRF Department of Medical Oncology St Bartholemew's Hospital London UK; 2 Sarcoma Unit Royal Marsden Hospital Fulham Road London SW3 6JJ UK; 3 Institute of Cancer Research Belmont UK

## Abstract

*Purpose*. The study was performed to assess the antitumour activity and toxicity of a 72-h continuous infusion of single-agent etoposide as second-line treatment for patients with locally advanced or metastatic soft tissue sarcoma (STS), following reports of substantial activity using this schedule of etoposide administration as first-line treatment in combination with ifosfamide.

*Patients/method*. This was an open phase I/II trial performed at a single institution in patients with metastatic or locally advanced STS who had failed first-line treatment with doxorubicin + ifosfamide combination chemotherapy or, less commonly, single-agent treatment with doxorubicin or ifosfamide. Etoposide was given as a continuous intravenous infusion over 72 h. The starting dose level was 200 mg m^-2^ day^-1^ × 3 escalating in 10% steps in cohorts of three patients until dose-limiting toxicity was encountered.

*Results*. Seventeen patients were treated, median age 47 years (range 26–71 years). No responses were seen in 16 assessable patients despite etoposide levels in the cotoxic range. The steady-state plasma concentration exceeded 8 *μ*g ml−^1^ in all patients and in patients treated at ≥ 600 mg m −^2^ the mean steady-state level was 14.4 *μ*g ml −^1^. The median event-free survival was 6 weeks (95% confidence interval (CI) 3.31–8.69) and the overall survival 16 weeks (95% CI 9.28–22.72). The maximum tolerated dose in this pretreated patient group was 200 mg mm^-2^ day^-1^ × 3. The dose-limiting toxicity was myelosuppression.

*Discussion*. Etoposide given by 72-h infusion is inactive as second-line chemotherapy in STS. It is associated with significant toxicity when given in these doses, in this patient group.


*Sarcoma* (1997) 1, 149–154

CARFAX

ORIGINAL ARTICLE

### A phase I/II study of a 72-h continuous infusion of etoposide in advanced soft tissue sarcoma

CHARLES R CRAWLEY,[1] IAN R. JUDSON,[2] MARK VERRILL,[2] CATHERINE HILL[2] & FLORENCE I. RAYNAUD[3]

[1]*ICRF Department of Medical Oncology, St Bartholemew's Hospital, London,* [2]*Sarcoma Unit, Royal Marsden Hospital, London &* [3]*Institute of Cancer Research, Belmont, UK*

**Abstract**

*Purpose.* The study was performed to assess the antitumour activity and toxicity of a 72-h continuous infusion of single-agent etoposide as second-line treatment for patients with locally advanced or metastatic soft tissue sarcoma (STS), following reports of substantial activity using this schedule of etoposide administration as first-line treatment in combination with ifosfamide.

*Patients/method.* This was an open phase I/II trial performed at a single institution in patients with metastatic or locally advanced STS who had failed first-line treatment with doxorubicin + ifosfamide combination chemotherapy or, less commonly, single-agent treatment with doxorubicin or ifosfamide. Etoposide was given as a continuous intravenous infusion over 72 h. The starting dose level was 200 mg $m^{-2}$ $day^{-1}$ $\times 3$ escalating in 10% steps in cohorts of three patients until dose-limiting toxicity was encountered.

*Results.* Seventeen patients were treated, median age 47 years (range 26–71 years). No responses were seen in 16 assessable patients despite etoposide levels in the cytotoxic range. The steady-state plasma concentration exceeded 8 $\mu$g $ml^{-1}$ in all patients and in patients treated at $\geqslant 600$ mg $m^{-2}$ the mean steady-state level was 14.4 $\mu$g $ml^{-1}$. The median event-free survival was 6 weeks (95% confidence interval (CI) 3.31–8.69) and the overall survival 16 weeks (95% CI 9.28–22.72). The maximum tolerated dose in this pretreated patient group was 200 mg $m^{-2}$ $day^{-1}$ $\times 3$. The dose-limiting toxicity was myelosuppression.

*Discussion.* Etoposide given by 72-h infusion is inactive as second-line chemotherapy in STS. It is associated with significant toxicity when given in these doses, in this patient group.

**Key words:** *continuous infusion, etoposide, sarcoma.*

### Introduction

Etoposide (VP-16-213) is a semi-synthetic derivative of podophyllotoxin. It first underwent clinical trials 20 years ago. It has significant activity in lymphoma, leukaemia and small cell lung cancer and is included in most standard regimens for the treatment of germ cell tumours. It acts primarily by inhibition of topoisomerase II resulting in double- and single-strand DNA breaks.[1]

The initial trials of etoposide as a single agent in the treatment of soft tissue sarcoma (STS), almost all carried out in pretreated patients, have been disappointing. Welt *et al.* reported a dose escalation study in pretreated patients with a 3-day alternating schedule.[2] The starting dose was 120 mg $m^{-2}$ given intravenously every other day for three doses, repeated every 3 weeks, increasing to a maximum of 240 mg $m^{-2}$ per dose. While toxicity was low, there was only one minor response, which included complete resolution of lung metastases, and 4/26 patients had stable disease. Similarly, a phase II EORTC study using a treatment schedule of 130 mg $m^{-2}$ po daily for 5 days every 3 weeks, in mostly heavily pretreated patients, reported only one partial response (PR), which lasted 19 months, in 29 patients.[3]

However, responses have been reported using more prolonged exposure. Hainsworth *et al.*[4] performed a phase I study with daily oral etoposide at 50 mg $mg^{-2}$ for 21 days. Seventeen patients with refractory disease were treated, there were five PRs lasting 4 months including 2/3 patients with STS. Kampe *et al.* investigated a more prolonged oral treatment regimen but reported no responses in 15 patients treated using 150 mg $m^{-2}$ po daily for 15 days. No patient received more than two courses.

Correspondence to: I. R. Judson, Sarcoma Unit, Royal Marsden Hospital, Fulham Road, London SW3 6JJ, UK. Tel: +44 (0)181 643 8901 ext 4302; Fax: +44 (0)181 770 7885; E-mail: judson@icr.ac.uk.

1357-714X/97/030149-06 $9.00 © 1997 Carfax Publishing Ltd

Three phase II studies performed using a schedule a schedule identical to that of Hainsworth *et al.*, i.e. 50 mg $m^{-2}$ $\times 21$ days every 28 days, failed to show significant activity.[6–8]

Etoposide has a schedule-dependent mechanism of action, as elegantly demonstrated by Slevin *et al.* in small cell lung cancer.[9] This study compared the activity of 5 daily 2-h infusions with the same total dose given as a continuous intravenous infusion over 24 h. The 5-day course was significantly superior with a response rate of 89% vs 10% despite the area under the concentration × time curve (AUC) being identical for both schedules. However, when treatment duration was extended to 8 days, no additional benefit was seen compared with 5 days,[10] and a 15-day infusion study had to be stopped early because of a worse response in the 15-day arm.[11] Thompson *et al.* performed a study using a protracted intravenous infusion of etoposide. This study, carried out in patients with potentially etoposide-sensitive malignancies, was performed at doses of 18–25 mg $m^{-2}$ $day^{-1}$ for 21–153 days.[12] The median duration of treatment was 17 weeks (range 3–80 weeks) and the overall response rate was 47%. The mean serum etoposide concentration was $0.7 \pm 0.42$ $\mu g$ $m^{-1}$ and antitumour activity was observed with levels from 0.5 to 1.0 $\mu g$ $m^{-1}$. This was lower than the effective level suggested by Clark *et al.*[10] in relation to small cell lung cancer, who reported that serum etoposide levels $>1$ $\mu g$ $m^{-1}$ were associated with antitumour activity and levels $>3$ $\mu g$ $m^{-1}$ with haematological toxicity. This is consistent with the model of a therapeutic window for etoposide with both the cytotoxicity and haematological toxicity thresholds falling as the duration of exposure increases.[13]

The Scandinavian Sarcoma Group reported a study of first-line chemotherapy in STS using etoposide by 72-h continuous infusion (200 mg $m^{-2}$ $day^{-1}$) plus ifosfamide 1.5 g $m^{-2}$ $day^{-1}$ by 2-h infusion daily $\times 3$.[14] They reported a PR rate of 40% in 33 patients with a median time to progression of 8 months. Confidence intervals (CIs) were not given but were presumably quite wide. Nevertheless, this level of activity suggests that etoposide may be active when given by 72-h infusion, given that response rates for single-agent ifosfamide at a standard dose of 5 g $m^{-2}$ have been in the region of 25%.[15] Evaluation of this schedule of etoposide as a single agent as second-line treatment in STS seemed warranted.

### Patients and methods

### *Eligibility criteria*

The following entry criteria were required: (1) histological evidence of STS; the following tumour types were excluded: Ewing's sarcoma, embryonal or alveolar rhabdomyosarcoma, osteosarcoma and malignant mesothelioma; (2) at least one bidimensionally measurable lesion with evidence of progression within 6 weeks prior to treatment, (3) white blood count (WBC) $\geqslant 3.0 \times 10^9$/l and platelets $\geqslant 100 \times 10^9$/l; (4) WHO performance status 0–2; (5) age 15–75 years; (6) expected prognosis $>12$ weeks; (7) effective contraception for both sexes; (8) informed consent and expected cooperation during follow-up. Prior treatment with doxorubicin and/or ifosfamide was allowed but not required. Exclusion criteria were: (1) radiotherapy to the sole index lesion; (2) known central nervous system metastases; (3) second primary malignant disease other than adequately treated *in situ* carcinoma of the cervix or basal or squamous cell carcinoma of the skin; (4) pregnant or lactating women. Written informed consent was obtained from all patients and the study was approved by the local Research Ethics Committee.

### *Treatment schedule*

Etoposide was given as a 72-h continuous intravenous (iv) infusion every 3 weeks. In this pretreated patient group, we intended to define the maximum tolerated dose (MTD) of etoposide for this schedule. Cohorts of three patients were treated at each dose level until the MTD was reached. Blood counts were performed weekly. Dose-limiting toxicity was defined as grade IV neutropenia for 7 or more days or grade IV thrombocytopenia of any duration. The maximum tolerated dose was defined as that dose causing dose-limiting toxicity in $\geqslant 60\%$ of patients. If one patient experienced grade IV neutropenia for more than 7 days or grade IV thrombocytopenia, then an additional two patients were treated at that dose level. If two or more patients experienced dose-limiting toxicity, then all subsequent patients were treated at the next lower dose level. The initial dose was set at 200 mg $m^{-2}$ $day^{-1}$ for 3 days; subsequent dose levels were to be 220 and 240 mg $m^{-2}$ $day^{-1}$. Steady-state plasma levels of etoposide were measured on days 2 and 3 of the infusion for the first cycle. In any patient, treatment was delayed for 1 week if the WBC was $<2.0 \times 10^9$/l, neutrophil count $<1.0 \times 10^9$/l or platelets $<100 \times 10^9$/l on the day treatment was due. If treatment had to be delayed by more than 1 week, then subsequent doses were reduced by 20%. If any treatment cycle was complicated by grade IV neutropenia and infection, then a similar dose reduction was employed.

### *Evaluation*

Pretreatment evaluation consisted of clinical history, physical examination, full blood count, urea and electrolytes and liver function tests, a chest radiograph and a computed tomography (CT) scan.

Repeat evaluations of known sites of disease were performed after two cycles of treatment and thereafter with alternate cycles of treatment. Event-free survival (EFS) was calculated from the date of study entry to the date of disease progression or death. Patients progressing during the first two cycles were considered as early progression. Toxicity was graded according to the National Cancer Institute common toxicity criteria (CTC). Responses were evaluated according to WHO criteria.[16] The duration of EFS and overall survival (OS) was estimated by the Kaplan–Meier method. Evidence of an association between plasma etoposide levels and haematological toxicity was assessed by the Pearson correlation coefficient (using the SPSS statistical package).

### *Measurement of plasma etoposide levels*

Steady-state plasma etoposide levels were measured using a high performance liquid chromatography (HPLC) method, as described by Harvey *et al.* using a phenytoin internal standard.[17] Calibration was achieved in plasma by the external calibration method using etoposide standards over the range 1–24 $\mu g\ m^{-1}$ vs an internal standard, phenytoin. Standards were prepared freshly for each HPLC run. Samples were extracted using dichloromethane, the organic phase was evaporated to dryness and reconstituted in methanol–water (51:49) and centrifuged at 300 $\times$ *g* before loading on to the autosampler.

### Results

A total of 17 patients were treated with a total of 44 cycles of chemotherapy (median 2, range 1–5). Patient characteristics are detailed in Table 1. All patients had received one line of prior chemotherapy, most commonly a combination of ifosfamide and doxorubicin as part of an EORTC study in which ifosfamide was given at 5 g $m^{-2}$, and doxorubicin at either 50 or 75 mg $m^{-2}$, the latter dose supported by granulocyte–macrophage colony-stimulating factor (BM-CSF). Single-agent doxorubicin was given to three patients at 75 mg $m^{-2}$ and a combination of doxorubicin with ifosfamide at 5–9 g $m^{-2}$ to two patients. Three patients were initially treated elsewhere, two received doxorubicin and one epirubicin. Two patients had received two prior chemotherapy regimens. All 17 patients were assessable for toxicity, 16 were assessable for response and one patient died due to neutropenia-related infection prior to any response assessment. One patient withdrew from the study after two courses with stable disease and subsequently received four more courses of etoposide off study. He currently remains alive with stable disease. The initial dose level was 200 mg $m^{-2}$ $day^{-1}$ for 3 days; four patients were treated at this dose before escalating to a dose level of 220 mg $m^{-2}$ $day^{-1}$ for 3 days. Five patients were treated at this dose but toxicity proved unacceptable and the remaining eight patients were treated at the lower dose level. Despite this, five patients required dose reductions (four at 200 mg $m^{-2}$ $day^{-1}$ and one at 220 mg $m^{-2}$ $day^{-1}$).

### *Response*

No responses were seen. Eight patients had stable disease and eight patients progressed through chemotherapy. The median EFS was only 6 weeks (95% CI 3.31–8.69) and the median OS was 16 weeks (95% CI 9.28–22.72) (Fig. 1). In the eight patients with stable disease the median progression-free survival was 3.5 months. Of the other two surviving patients, one subsequently received abdominal radiotherapy resulting in a PR. She has subsequently progressed with hepatic metastases, while the other has had no further therapy.

### *Plasma etoposide concentrations*

Data were available on 12 patients and 70 courses. Where steady-state levels at two time points during

**Table 1.** *Patient characteristics*

| Parameter | Number of patients |
|---|---|
| Registered | 17 |
| Assessable for response | 16 |
| Assessable for toxicity | 17 |
| Age | |
| Median | 47 |
| Range | 26–71 |
| Sex | |
| Male | 10 |
| Female | 7 |
| Performance status (WHO) | |
| 0 | 1 |
| 1 | 12 |
| 2 | 4 |
| Histology | |
| Leiomyosarcoma | 8 |
| Liposarcoma | 3 |
| Malignant peripheral nerve sheath | 2 |
| Synovial sarcoma | 1 |
| Malignant fibrous histiocytoma | 1 |
| Uterine leiomyosarcoma | 1 |
| High grade not classified | 1 |
| Grade | |
| Low | 6 |
| Intermediate | 1 |
| High | 8 |
| Not known | 2 |
| Prior chemotherapy | 17 |
| Response to prior chemotherapy | |
| Progression | 3 |
| Stable disease | 11 |
| Partial response | 2 |

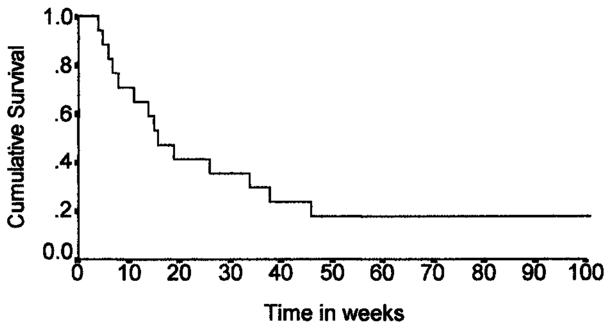

**Fig. 1.** *Overall survival in patients with recurrent STS treated with 72-h etoposide.*

the 72-h infusion were measured, the mean plasma level has been calculated (16/20). The results are shown in Tables 2 and 3. All concentrations were above those previously reported as the cytotoxic threshold.[11,12] There was no clear association between plasma level and CTC grade haematological toxicity (Fig. 2) ($r = 0.29$, $p = 0.26$) and there was no significant correlation between dose and mean plasma level ($r = 0.28$, $p = 0.24$) (Table 2).

*Toxicity*

Toxicity was significant and primarily haematological. Data were available on 17 patients and for 95% of the treatment cycles. The majority, i.e. 76%, of patients experienced grade IV neutropenia. Thrombocytopenia was less of a problem with grade IV toxicity in only one patient. Blood transfusions were required in 47% of patients. Growth factor support

**Table 2.** *Plasma etoposide concentration*

| Patient | Treatment cycle | Dose ($mg\ m^{-2}$) | Mean etoposide level ($mg\ ml^{-1}$) |
|---|---|---|---|
| 1 | 2 | 480 | 8.7 |
| 2 | 2 | 528 | 9.3 |
| 3 | 1 | 600 | 8.3 |
| 4 | 1 | 600 | 13.9 |
| 4 | 2 | 600 | 12.3 |
| 5 | 1 | 600 | 16.8 |
| 6 | 1 | 600 | 20.0 |
| 6 | 3 | 600 | 23.1 |
| 7 | 2 | 600 | 8.8 |
| 8 | 2 | 600 | 22.9 |
| 9 | 1 | 600 | 9.2 |
| 10 | 2 | 600 | 11.8 |
| 10 | 3 | 600 | 14.3 |
| 10 | 1 | 600 | 14.4 |
| 11 | 1 | 600 | 11.5 |
| 11 | 1 | 660 | 8.3 |
| 11 | 3 | 660 | 21.6 |
| 12 | 1 | 660 | 14.1 |
| 12 | 1 | 660 | 14.2 |
| 12 | 2 | 660 | 14.1 |

**Table 3.** *Mean etoposide concentration vs dose*

| Dose ($mg\ m^{-2}$) | Mean concentration ($\mu g\ ml^{-1} \pm SD$) |
|---|---|
| < 600 | 9.0 (0.4) |
| 600 | 14.4 (4.2) |
| 660 | 14.5 (4.7) |

(Correlation coefficient $r = 0.28$, $p = 0.24$).

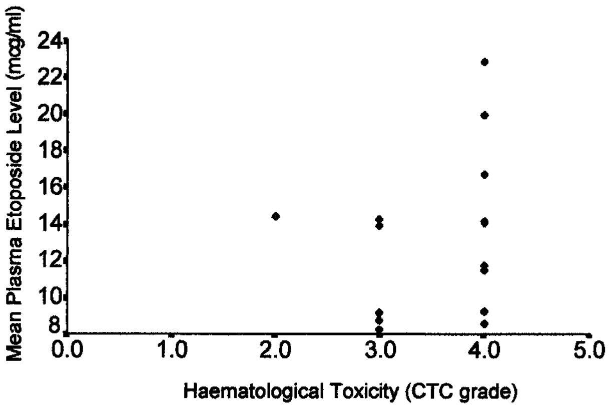

**Fig. 2.** *Haematological toxicity vs mean plasma etoposide levels (correlation coefficient* r = 29, p = 0.26).

was not used. Three patients died on treatment, one from *Escherichia coli* sepsis when neutropenic, one at home from an acute abdominal catastrophe during her third course, her disease having been stable after the second course. She had a normal blood count at the time of the last chemotherapy 6 days before her death. The third patient died due to progressive pulmonary disease but was neutropenic at the time of his death. Early progression was seen in two additional patients who both received only one course of treatment. Seven patients were admitted for neutropenic sepsis. Other admissions (total three) were for problems related to disease progression. Non-haematological toxicity was less of a problem. Two patients (11%) experienced grade III emesis and one patient developed pulmonary oedema due to the fluid load. Alopecia was almost universal but mostly predated etoposide The remaining toxicities were relatively mild and included mucositis in seven patients (grade 1–2), diarrhoea in three patients (grade 2), fatigue and asymptomatic elevation of liver enzymes.

### Discussion

In this study, 17 patients with recurrent STS were treated with a continuous infusion of etoposide. A dose escalation was planned but was not feasible. The maximum tolerated dose in this pretreated patient group was 600 mg $m^{-2}$. Plasma etoposide concentrations showed considerable inter-patient variation (range 8.3–22.9 $\mu g\ m^{-1}$, for patients receiving 600 mg $m^{-2}$). We were unable to demonstrate a correlation between neutropenia and plasma level; however, our starting dose was at the MTD and 76% of cycles were associated with grade IV neutropenia. The numbers were insufficient to show a correlation between plasma concentration and dose. Overall, the toxicity was significant with three deaths on treatment. In the 16 assessable patients, no responses were seen, although stable disease was observed in eight patients for short periods. One patient had stable disease for more than 12 months.

These results are disappointing. Given the lack of activity of etoposide in either IV bolus or protracted oral schedules,[2,4,6,8] the lack of demonstrable activity for etoposide in this schedule is likely to present drug resistance rather than an inactive schedule. In this setting, it seems unlikely that dose escalation with cytokine support will produce useful responses or effective palliation.

It is not possible to determine whether this represents *de novo* drug resistance or acquired resistance after previous doxorubicin/ifosfamide exposure. Of note, one patient who progressed on etoposide subsequently had a transient response to high-dose ifosfamide (12 g $m^{-2}$). It should be noted that this patient group had unusually refractory disease to first-line chemotherapy with doxorubicin and/or ifosfamide chemotherapy with a response rate of only 12.5% (2/16). This is outside the 95% CI for doxorubicin alone, doxorubicin/ifosfamide or CYVADIC in the randomized EORTC study which compared these three lines of treatment.[18]

In summary, there is little evidence of activity of etoposide in any schedule in relapsed or progressive STS. This study shows that a 72-h etoposide infusion at the maximum tolerated dose is inactive in previously treated patients in common with other reports using slightly different schedules. The 40% response rate obtained by the Scandinavian Sarcoma Group[14] with ifosfamide and etoposide as first-line treatment may reflect genuine etoposide activity in previously untreated patients or, alternatively, a schedule advantage for fractionated ifosfamide treatment given that the ifosfamide was given as three consecutive daily infusions. A recent randomized phase II study performed by the EORTC Soft Tissue and Bone Sarcoma Group demonstrated a markedly higher response rate when ifosfamide, albeit at a higher total dose, was given as three daily infusions of 3 g $m^{-2}$ compared with a single 24-h infusion of 5 g $m^{-2}$, i.e. 17% vs 3%.[19] Both response rates were lower than expected for reasons which are not clear. However, the response rate in the Scandinavian study remains within the range of reported response rates for phase II studies of ifosfamide alone.[14] There is no indication for the use of etoposide in any regimen in pretreated patients and its inclusion in combination regimens as initial therapy should be evaluated in prospective randomized trials.

### References

1 van Maanen JM, Retel J., de Vries J, *et al.* Mechanism of action of antitumor drug etoposide: a review. *J Natl Cancer Inst* 1988; 80:1526–33.

2 Welt S, Magill GB, Sordillo PP, *et al.* Phase II trial of VP-16-213 in adults with advanced soft tissue sarcomas. *Proc Am Soc Clin Oncol* 1983; 3:234 (Abstract).

3 Dombernowski P, Buesa J, Pinedo HM, *et al.* VP-16 in advanced soft tissue sarcoma: a phase II study of the EORTC soft tissue and bone sarcoma group. *Eur J Cancer Clin Oncol* 1987; 23:579–80.

4 Hainsworth JD, Johnson DH, Frazier SR, *et al.* Chronic daily administration of oral etoposide-a phase I trial. *J Clin Oncol* 1989; 7:396–401.

5 Kampe CE, Lowenbraun S, Foster J, *et al.* Oral etoposide in treatment of advanced refractory sarcomas. *J Natl Cancer Inst* 1992; 84:1836–7.

6 Licht JD, Mazanet R, Loehrer PJ, *et al.* Phase IV trial of daily oral etoposide in the treatment of advanced soft-tissue sarcoma. *Cancer Chemother Pharmacol* 1994; 34:79–80.

7 Linke KA, Hays C, Burgess MA, *et al.* Oral etoposide (VP-16) results in previously treated soft tissue sarcoma patients. *Proc Am Assoc Cancer Res* 1993; 34:208.

8 Keizer HJ, Crowther D, Steen Nielsen O, *et al.* EORTC Group phase II study of oral etoposide for pretreated soft tissue sarcoma. *Sarcoma* 1997; 1:99–101.

9 Slevin ML, Clark PI, Joel SP, *et al.* A randonlized trial to evaluate the effect of schedule on the activity of etoposide in small-cell lung cancer. *J Clin Oncol* 1989; 7:1333–40.

10 Clark PI, Slevin ML, Joel SP, *et al.* A randomized trial of two etoposide schedules in small-cell lung cancer: the influence of pharmacokmetics on efficacy and toxicity. *J Clin Oncol* 1994; 12:1427–35.

11 Joel SP, O'Bryan K, Penson R, *et al.* A randomised, concentration-controlled, comparison of standard (5 day) vs prolonged (15 day) infusions of etoposide in small cell lung cancer (SCLC). *Proc Am Soc Clin Oncol* 1997; 16:451a (Abstract 1622).

12 Thompson DS, Hainsworth JD, Hande KR, *et al.* Prolonged administration of low-dose, infusional etoposide in patients with etoposide-sensitive neoplasms: a phase I/II study. *J Clin Oncol* 1993; 11:1322–8.

13 Joel SP. The clinical pharmacology of etoposide: an update. *Cancer Treat Rev* 1997; 22:179–221.

14 Saeter, G, Talle K, Solheim OP. Treatment of advanced, high-grade soft-tissue sarcoma with ifosfamide and continuous-infusion etoposide. *Cancer Chemother Pharmacol* 1995; 36:172–5.

15 Bramwell VH, Mouridsen HT, Santoro A, *et al.* Cyclophosphamide versus ifosfamide: a randomized phase II trial in adult soft-tissue sarcomas. The European Organization for Research and Treatment of Cancer, Soft Tissue and Bone Sarcoma Group. *Cancer Chemother Pharmacol* 1993; 31 Suppl. 2:S180–4.

16 Miller AB, Hoogstraten B, Staquet M, *et al.* Reporting results of cancer treatment. *Cancer* 1981; 47:207–14.

17 Harvey VJ, Joel SP, Johnston A, *et al.* High performance liquid chromatography of etoposide in plasma and urine. *J Chromatography* 1985; 339:419–23.

18 Santoro A, Tursz T, Mouridsen H. *et al.* Doxorubicin versus CYVADIC versus doxorubicin plus ifosfamide in first-line treatment of advanced soft tissue sarcomas: a randomized study of the European Organization for Research and Treatment of Cancer Soft Tissue and Bone Sarcoma Group. *J Clin Oncol* 1995; 13:1537–45.

19 van Oosterom AT, Krzemienlecki K, Nielsen OS, *et al.* Randomised phase II study of the EORTC Soft-Tissue and Bone Sarcoma (STBSG) group comparing two different ifosfamide (IF) regimens in chemotherapy untreated advanced soft tissue sarcoma (STS) patients (pts). *Proc Am Soc Clin Oncol* 1997; 16:496a (Abstract 1787).

## References

[PDF_00535] Bramwell V. H., Mouridsen H. T., Santoro A., Blackledge G., Somers R., Verweij J., Dombernowsky P., Onsrud M., Thomas D., Sylvester R. (1993). Cyclophosphamide versus ifosfamide: a randomized phase II trial in adult soft-tissue sarcomas. The European Organization for Research and Treatment of Cancer [EORTC], Soft Tissue and Bone Sarcoma Group.. Cancer Chemother Pharmacol.

[PDF_00515] Clark P. I., Slevin M. L., Joel S. P., Osborne R. J., Talbot D. I., Johnson P. W., Reznek R., Masud T., Gregory W., Wrigley P. F. (1994). A randomized trial of two etoposide schedules in small-cell lung cancer: the influence of pharmacokinetics on efficacy and toxicity.. J Clin Oncol.

[PDF_00490] Dombernowsky P., Buesa J., Pinedo H. M., Santoro A., Mouridsen H., Somers R., Bramwell V., Onsrud M., Rouesse J., Thomas D. (1987). VP-16 in advanced soft tissue sarcoma: a phase II study of the EORTC soft tissue and bone sarcoma group.. Eur J Cancer Clin Oncol.

[PDF_00494] Hainsworth J. D., Johnson D. H., Frazier S. R., Greco F. A. (1989). Chronic daily administration of oral etoposide--a phase I trial.. J Clin Oncol.

[PDF_00543] Harvey V. J., Joel S. P., Johnston A., Slevin M. L. (1985). High-performance liquid chromatography of etoposide in plasma and urine.. J Chromatogr.

[PDF_00529] Joel S. (1996). The clinical pharmacology of etoposide: an update.. Cancer Treat Rev.

[PDF_00497] Kampe C. E., Lowenbraun S., Foster J., Rosen G. (1992). Oral etoposide in treatment of advanced refractory sarcomas.. J Natl Cancer Inst.

[PDF_00508] Keizer H. J., Crowther D., Nielsen O. S., Oosterom A. T., Muguiro J. H., Pottelberghe C. V., Somers R., Tursz T. (1997). EORTC Group Phase II Study of Oral Etoposide for Pretreated Soft Tissue Sarcoma.. Sarcoma.

[PDF_00500] Licht J. D., Mazanet R., Loehrer P. J., Gonin R., Antman K. H. (1994). Phase IV trial of daily oral etoposide in the treatment of advanced soft-tissue sarcoma.. Cancer Chemother Pharmacol.

[PDF_00541] Miller A. B., Hoogstraten B., Staquet M., Winkler A. (1981). Reporting results of cancer treatment.. Cancer.

[PDF_00531] Saeter G., Talle K., Solheim O. P. (1995). Treatment of advanced, high-grade soft-tissue sarcoma with ifosfamide and continuous-infusion etoposide.. Cancer Chemother Pharmacol.

[PDF_00546] Santoro A., Tursz T., Mouridsen H., Verweij J., Steward W., Somers R., Buesa J., Casali P., Spooner D., Rankin E. (1995). Doxorubicin versus CYVADIC versus doxorubicin plus ifosfamide in first-line treatment of advanced soft tissue sarcomas: a randomized study of the European Organization for Research and Treatment of Cancer Soft Tissue and Bone Sarcoma Group.. J Clin Oncol.

[PDF_00511] Slevin M. L., Clark P. I., Joel S. P., Malik S., Osborne R. J., Gregory W. M., Lowe D. G., Reznek R. H., Wrigley P. F. (1989). A randomized trial to evaluate the effect of schedule on the activity of etoposide in small-cell lung cancer.. J Clin Oncol.

[PDF_00524] Thompson D. S., Hainsworth J. D., Hande K. R., Holzmer M. C., Greco F. A. (1993). Prolonged administration of low-dose, infusional etoposide in patients with etoposide-sensitive neoplasms: a phase I/II study.. J Clin Oncol.

[PDF_00484] van Maanen J. M., Retèl J., de Vries J., Pinedo H. M. (1988). Mechanism of action of antitumor drug etoposide: a review.. J Natl Cancer Inst.

